# Copy number variants are a common cause of non-syndromic hearing loss

**DOI:** 10.1186/gm554

**Published:** 2014-05-22

**Authors:** A Eliot Shearer, Diana L Kolbe, Hela Azaiez, Christina M Sloan, Kathy L Frees, Amy E Weaver, Erika T Clark, Carla J Nishimura, E Ann Black-Ziegelbein, Richard J H Smith

**Affiliations:** 1 Department of Otolaryngology - Head and Neck Surgery, Molecular Otolaryngology & Renal Research Labs, University of Iowa Hospitals and Clinics, Iowa City, Iowa 52242, USA; 2 Iowa Institute of Human Genetics, University of Iowa College of Medicine, Iowa City, Iowa 52242, USA; 3 Interdepartmental PhD Program in Genetics, University of Iowa, Iowa City, Iowa 52242, USA

## Abstract

**Background:**

Copy number variants (CNVs) are a well-recognized cause of genetic disease; however, methods for their identification are often gene-specific, excluded as ‘routine’ in screens of genetically heterogeneous disorders, and not implemented in most next-generation sequencing pipelines. For this reason, the contribution of CNVs to non-syndromic hearing loss (NSHL) is most likely under-recognized. We aimed to incorporate a method for CNV identification as part of our standard analysis pipeline and to determine the contribution of CNVs to genetic hearing loss.

**Methods:**

We used targeted genomic enrichment and massively parallel sequencing to isolate and sequence all exons of all genes known to cause NSHL. We completed testing on 686 patients with hearing loss with no exclusions based on type of hearing loss or any other clinical features. For analysis we used an integrated method for detection of single nucleotide changes, indels and CNVs. CNVs were identified using a previously published method that utilizes median read-depth ratios and a sliding-window approach.

**Results:**

Of 686 patients tested, 15.2% (104) carried at least one CNV within a known deafness gene. Of the 38.9% (267) of individuals for whom we were able to determine a genetic cause of hearing loss, a CNV was implicated in 18.7% (50). We identified CNVs in 16 different genes including 7 genes for which no CNVs have been previously reported. CNVs of *STRC* were most common (73% of CNVs identified) followed by CNVs of *OTOA* (13% of CNVs identified).

**Conclusion:**

CNVs are an important cause of NSHL and their detection must be included in comprehensive genetic testing for hearing loss.

## Background

Copy number variants (CNVs) are genomic variants that alter the diploid state of a portion of the genome, either by increasing (duplications, triplications) or decreasing (deletions) the number of alleles. CNVs range in size from 50 bp, the size of a small exon, to 5 megabases, the limit of detection for cytogenetic microscopy; they therefore span the continuum of genetic variation between small insertions and deletions (indels) to large chromosomal alterations [1]. CNVs can be benign or pathogenic, with CNV-induced pathogenesis typically due to 1) changes in copy number of dosage-sensitive genes, 2) gene disruption, or 3) fusion events leading to novel genes [2].

Whereas the mechanism of formation of single nucleotide mutations or indels is errors of DNA replication and repair, genomic rearrangements that produce CNVs are driven primarily by genomic architecture [2]. CNVs can be categorized by mechanism of formation, which in turn defines characteristics specific to CNVs, such as breakpoints and recurrence likelihood. Three mechanisms of formation have been described and shown experimentally to account for the vast majority of CNVs in the human genome: 1) non-allelic homologous recombination (NAHR), caused by segmental duplications or repetitive elements such as Alu or L1 repeats and leading to recurrent CNVs, 2) non-homologous end-joining (NHEJ), mediated in some instances by repetitive elements and leading to non-recurrent CNVs, and 3) fork stalling and template switching (FoSTeS), leading to non-recurrent CNVs (reviewed in [2]).

The growing appreciation of the importance CNVs in human disease reflects our better understanding of their formation and improved tools for their identification. The Human Gene Mutation Database contains 148,413 reported disease-causing mutations in 6,137 genes [3] (accessed February 2014). Of these mutations, 89.6% are single nucleotide changes and small (< 20 bp) indels. The remaining 15,072 (11.4%) are structural variants that include 10,968 deletions, 2,600 insertions, and 1,504 complex rearrangements. Examples of the contribution of CNVs to human genetic disease include Charcot-Marie-Tooth neuropathy 1A (autosomal dominant), Gaucher disease (autosomal recessive), hemophilia A (X-linked) and mental retardation (complex genetics) (reviewed in [4]).

CNVs have also been identified as a cause of non-syndromic hearing loss (NSHL), the most well-known example being deletion of a segmental duplication region of chromosome 15 that includes the gene *STRC* and causes autosomal recessive NSHL (ARNSHL) at the DFNB16 locus [5,6] or deafness-infertility syndrome (DIS) if the adjacent *CATSPER2* gene also is involved [7]. Amongst the 89 genes involved in NSHL (this list includes genes that cause syndromic hearing loss that can mimic NSHL), CNVs have been reported in 18 (Table 1). To date, however, there have been more than 1,000 reported causative single nucleotide variants and indels in these same genes [8].

**Table 1:** CNVs identified to date in non-syndromic hearing loss genes and non-syndromic hearing loss mimic genes

**Gene**	**Phenotype**	**Locus**	**CNV type**	**CNV size**	**Pubmed ID**
*ALMS1*	AS	-	Deletion	Partial gene deletion	This study
*DFNA5*	ADNSHL	DFNA5	Complex	1.2 kb deletion with 127 bp insertion	9771715
*EYA4*	DCM + NSHL, ADNSHL	DFNA10	Deletion/duplication	Partial gene deletion/partial gene duplication	15735644, this study
*GJB2*	ARNSHL, ADNSHL	DFNB1/DFNA3	Deletion	Partial, whole gene, and upstream regulatory region deletion	19101659, 20236118, 15994881
*GJB6*	ARNSHL, ADNSHL	DFNB1/DFNA3	Deletion	Partial, whole gene, and upstream regulatory region deletion	11896458, 11807148, 11668644, this study
*MYH9*	MYH9-Disease, ADNSHL	DFNA17	Deletion/duplication	Partial and whole gene deletion/partial gene duplication	18284620, this study
*MYO6*	ARNSHL, ADNSHL	DFNB37/DFNA22	Deletion	Partial gene deletion	This study
*MYO7A*	USH1, ARNSHL, ADNSHL	DFNB2/DFNA11/USH1B	Deletion	Partial gene deletion	9382091
*OTOA*	ARNSHL	DFNB22	Deletion/duplication/conversion	Partial or whole gene deletion/partial gene duplication/pseudogene conversions	19888295, this study
*OTOF*	ARNSHL	DFNB9	Deletion	Partial gene deletion	20211493
*PCDH15*	USH1, ARNSHL	DFNB23/USH1F	Deletion/duplication	Partial gene deletion/partial gene duplication	20538994, 16679490, 17277737
*PDZD7*	USH2 modifier	-	Deletion	Partial gene deletion	This study
*PNPT1*	ARNSHL	DFNB70	Duplication	Partial gene duplication	This study
*POU3F4*	XLNSHL	DFNX3	Deletion/complex rearrangements	Deletions and rearrangements of upstream regulatory regions	20412083, 16365218, 8872461, 19930154, 20668882
*SERPINB6*	ARNSHL	-	Deletion	Partial gene deletion	This study
*SLC26A4*	PDS, ARNSHL	DFNB4/PDS	Deletion	Partial gene deletion	17443271, 18285825, 19287372, 19287372, 12676893, this study
*STRC*	ARNSHL, DIS	DFNB16	Deletion/duplication/conversion	Whole gene deletions/whole gene duplications/pseudogene conversions	11687802, 17098888, this study
*TECTA*	ARNSHL	DFNB21/DFNA8/DFNA12	Deletion	Partial gene deletion	17431902
*TJP2*	ADNSHL	DFNA51	Duplication	Tandem inverted duplication of entire gene	20602916
*TMC1*	ARNSHL	DFNB7/DFNB11/DFNA36	Deletion	Partial gene deletion	11850618, 19187973, this study
*TMPRSS3*	ARNSHL	DFNB8/DFNB10	Deletions/complex	Partial gene deletion/complex microsatellite insertion	11137999, this study
*TRIOBP*	ARNSHL	DFNB28	Deletion	Whole gene deletion	This study
*USH1C*	USH1/hyperinsulism/enteropathy syndrome	DFNB18/USH1C	Deletion	Whole gene deletion	10973248
*USH2A*	USH2	-	Deletion	Partial gene deletion	This study
*WFS1*	DIDMOAD, ADNSHL	DFNA6/DFNA14	Deletion/duplication	Partial gene deletion/partial gene duplication	15277431, this study

*Abbreviations:ADNSHL*, autosomal dominant nonsyndromic hearing loss; ARNSHL, autosomal recessive nonsyndromic hearing loss; AS, Alström syndrome; DCM, dilated cardiomyopathy; DIDMOAD, aka Wolfram syndrome, diabetes insipidus, diabetes mellitus, optic atrophy, and deafness - autosomal recessive; DIS, deafness-infertility syndrome - autosomal recessive; PDS, Pendred syndrome, deafness, inner ear abnormalities, and thyroid dysfunction - autosomal recessive; USH, Usher syndrome - autosomal recessive; XLNSHL, X-linked nonsyndromic hearing loss.

We suspected that CNVs are an under-recognized cause of deafness, a limitation associated with the traditional low throughput and low-resolution methods for identification of genomic alterations. Classic methods for CNV identification include: 1) karyotyping and fluorescence *in situ* hybridization (FISH), a method limited to the resolution of a microscope; 2) array comparative genomic hybridization (array-CGH), a commonly used ‘high resolution’ CNV detection method that provides resolution to about 100 kb; and 3) assays such as multiplex ligation-dependent amplification (MLPA), customized array-CGH panels, and breakpoint mapping. While the resolution of these last methods is outstanding, their implementation is costly, time-consuming, and not scalable to the genome level. Additionally, these methods all rely on an isolated test for CNVs. The ideal genetic test should combine single nucleotide variant detection, indel detection and CNV identification. In this study we sought to determine the relative importance of CNVs as a cause of hearing loss through the use of a diagnostic platform that allows detection of all three of these types of variants simultaneously.

Targeted genomic enrichment coupled with massively parallel sequencing (TGE-MPS) has revolutionized clinical genetic testing by enabling specific genomic regions to be isolated, enriched and sequenced. TGE-MPS has been successfully applied to the diagnosis of several inherited diseases including deafness [9-11]. Following TGE, MPS generates millions of short sequencing reads that are mapped to the human reference genome to cover each sequenced nucleotide hundreds to thousands of times (referred to as fold-coverage). Variations from the reference genome are identified and annotated, and by capitalizing on sequencing depth of coverage between samples, alterations to the normal diploid genomic state can be identified and changes in copy number recognized (reviewed in [12]). Several groups, including ours, have successfully used these methods for identification of CNVs in patients with deafness and other genetic disorders [10,13-15].

In this study we use TGE-MPS with integrated CNV detection as part of a comprehensive clinical diagnostic platform for hearing loss. We performed genetic testing on 686 patients with hearing loss and we identified CNVs in 16 deafness-causing genes, including 7 genes in which CNVs have heretofore not been described. We show that CNVs are a major contributor to hereditary hearing loss, comprising nearly one in five of all diagnoses for NSHL. These data mandate the inclusion of CNV detection as standard on all platforms used for the clinical diagnosis of genetic deafness.

## Methods

### Subjects

Records were examined for all patients who underwent clinical genetic testing for deafness at our laboratory over a two-year period beginning in January 2012. Testing was completed using the TGE-MPS panel we have developed called OtoSCOPE®. We included only unique probands by excluding familial testing and repeat testing. No patients were excluded based on age, age of onset of hearing loss, previous testing, or type of hearing loss. This study was approved by the Institutional Review Board of the University of Iowa; because it was a retrospective review of clinical data with a limited chance for harm to patients, informed consent was not obtained and instead the study was granted a full HIPAA waiver of authorization. To ensure anonymity of patients in this retrospective clinical study, patient information was de-identified, including providing ages in ranges and not identifying the sex of the patient. Our ethical approval did not allow deposition of patient data in to a public repository. This research was performed in accordance with the Declaration of Helsinki.

### Library preparation, sequencing, and bioinformatics

TGE-MPS was completed as previously described (see [9] for details), using 3 μg of high-quality genomic DNA. Liquid-handling automation equipment was used to prepare the majority of libraries. We used OtoSCOPE v4 or v5, targeting 66 or 89 deafness-associated genes, respectively. We used v4 for 76 and v5 for 28 of the patients for whom we found CNVs, respectively (Additional file 1). We include all NSHL genes known at the time each respective version of OtoSCOPE was made (v4 designed May 2011, and v5 designed November 2012) as well as genes that cause syndromic forms of deafness that mimic NSHL at an early age. In addition to the inclusion of newly discovered NSHL genes, OtoSCOPE v5 also includes several more NSHL mimics and additional probe coverage over the extended *OTOA* genomic region as well as the *STRC* genomic region to improve our ability to delimit the size of copy number variants at these loci. We also added probes to cover the gene *CATSPER2*, which is upstream of *STRC* and often involved in CNVs at this locus.

Sequencing was performed in pools of up to 48 samples per Illumina HiSeq flow cell using 100-bp paired-end reads. Sample quality control values were maintained as described previously [9]. All samples either met these requirements or were re-run.

Data were analyzed using a local installation of the open-source Galaxy software [16,17] and a combination of several other open-source tools, including read mapping with Burrows-Wheeler Alignment (BWA) [18], duplicate removal with Picard, local re-alignment and variant calling with GATK Unified Genotyper [19], enrichment statistics with NGSRich [20], and reporting and annotation of variants with custom software [9].

For copy number analysis, we completed read mapping, duplicate removal, and re-alignment as described above. Next we used the indexed BAM file to call variants and generate a pileup using samtools [21] mpileup –Bf, retaining only the position and depth information for all variants called. We then performed CNV calling using a previously published tool written in R [22]. This method normalizes read-depth data by sample batch and compares median read-depth ratios using a sliding-window approach [22]. This tool is available by request from the original author (A Nord, personal communication). We performed this analysis using the 2011-07-01 version of the tool with default settings. CNV calls were curated through manual inspection.

We validated this method using MLPA spanning the *STRC* gene region on chromosome 15q15.3 [23]. Our validation set of samples included DNA from 60 *GJB2* negative probands with NSHL and 4 positive control individuals simultaneously tested with TGE-MPS and blinded MLPA testing. Results showed four homozygous deletions and four heterozygous deletions of this region with 100% concurrence between the methods.

All variant calls, including SNVs, indels and CNVs, were discussed at an interdisciplinary meeting (Hearing Group Meeting) that includes physicians, geneticists, genetic counselors, scientists and bioinformaticians. At these meetings, all available clinical, phenotypic and genetic data are used to determine the most likely genetic cause, if any, for hearing loss.

## Results

### Copy number variants identified

We identified 143 CNVs in 16 genes in 686 patients with hearing loss (Table 2; Additional file 2). Of the patients studied, 15.2% (104/686) carried at least one CNV in a known deafness gene. We report CNVs in 7 of these genes for the first time, increasing the number of known deafness genes with CNVs by 28% to 25 total genes (Table 1). The overall CNV carrier frequency was 20.8% (143 CNVs/686 patients). We identified a probable genetic cause of hearing loss, be it a single nucleotide variant, indel, or CNV, in 38.9% of patients enrolled in this study (267/686). Detailed diagnostic results for these patients not pertaining to CNVs will be reported elsewhere.

**Table 2:** Summary of all CNVs identified in 686 individuals requesting genetic testing for deafness using a comprehensive genetic testing platform

**Gene**	**Total CNVs**	**Carrier CNVs**	**Causative CNVs (%)**	**Deletions (%)**	**Conversions (%)**	**Duplications (%)**
*STRC*	105 (73%)	35 (61%)	70 (81%)	65 (71%)	35 (92%)	5 (38%)
*OTOA*	18 (13%)	11 (19%)	7 (8%)	12 (13%)	3 (8%)	3 (23%)
*GJB6*	4 (3%)	1 (2%)	3 (3%)	4 (4%)	-	-
*USH2A*	3 (2%)	2 (4%)	1 (1%)	2 (2)%	-	1 (8%)
*MYH9*	2 (1%)	1 (2%)	1 (1%)^a^	1 (1%)	-	1 (8%)
*ALMS1*	1 (1%)	1 (2%)	-	1 (1%)	-	-
*MYO6*	1 (1%)	1 (2%)	-	1 (1%)	-	-
*PDZD7*	1 (1%)	1 (2%)	-	1 (1%)	-	-
*SERPINB6*	1 (1%)	1 (2%)	-	1 (1%)	-	-
*SLC26A4*	1 (1%)	-	1 (1%)	1 (1%)	-	-
*TMC1*	1 (1%)	-	1 (1%)	1 (1%)	-	-
*TMPRSS3*	1 (1%)	-	1 (1%)	1 (1%)	-	-
*TRIOBP*	1 (1%)	-	1 (1%)^a^	1 (1%)	-	-
*EYA4*	1 (1%)	1 (2%)	-	-	-	1 (8%)
*WFS1*	1 (1%)	1 (2%)	-	-	-	1 (8%)
*PNPT1*	1 (1%)	1 (2%)	-	-	-	1 (8%)
**Total**	143	57	86	92	38	13

See Additional file 2 for details. ^a^Part of a large CNV encompassing at least 1.5 MB (see text for details).

Eighty-six CNVs were identified and considered causative in 18.7% (50/267) of diagnosed patients. Of these patients, the causative CNVs were categorized as follows: 21 (42%) homozygous CNVs (all deletions), 16 (32%) hemizygous CNVs found in conjunction with a second pathogenic change, 12 (24%) biallelic CNVs, and 2 (4%) CNVs that are part of a large heterozygous contiguous deletion of chromosome of at least 1.5 Mb on chromosome 22q12.3-22q13.1 in one patient (Additional file 2). Homozygous CNVs are defined as the same change to copy number on both alleles while biallelic mutations are defined as a different copy number change on each allele (that is, a gene deletion and a gene conversion).

Of 50 patients with causative CNVs, 48 were diagnosed with ARNSHL. Two patients segregated apparent autosomal dominant NSHL (ADNSHL), one of whom carried both a single nucleotide mutation in *DIAPH1* predicted to be pathogenic as well as a large heterozygous deletion of chromosome 22q12.3-22q13.1 of approximately 1.5 Mb that includes both *MYH9* and *TRIOBP*. The extent of this deletion could not be resolved further due to the lack of other targeted genes on this chromosome. In this patient either the large CNV or the mutation in *DIAPH1* may be responsible for the hearing loss. The size of the deletion and the absence of additional phenotypic data make a more definitive interpretation of these variants difficult. A subtle syndromic form of deafness is also possible. This patient was the only case in which two possible deafness-causing variants were identified.

The second patient segregating ADNSHL had moderate hearing loss and an out-of-frame deletion of the last 12 exons of *MYO6* predicted to be causative (Additional file 2). A deletion in *TMC1*, which can cause both ARNSHL and ADNSHL at the DFNB7/11 and DFNA36 loci, respectively, was considered pathogenic *in trans* with a second missense variant in a patient with ARNSHL. The only other CNV present in a gene implicated in ADNSHL was an in-frame duplication of exons 18 to 20 of *EYA4*. Mutations in *EYA4* cause ADNHSL at the DFNA10 locus. For this study we categorized this CNV as non-pathogenic, although further study is required to determine whether this CNV is translated and pathogenic through a dominant-negative effect.

Most CNVs were deletions (92, 64.3%), followed by gene conversions (38, 26.6%), and duplications (13, 9.1%) (Table 2). The CNVs we detected ranged in size from one exon to over 1.5 Mb (chromosome 22q12.3-22q13.1).

### The genomic architecture of hearing loss genes

To define the landscape of repeat elements that surround known NSHL genes, we performed a bioinformatics analysis of segdups (segmental duplications), Alu elements and L1 elements as these elements are sites of NAHR and mediate NHEJ [2]. Using data from RepeatMasker [24] we examined the presence of these repetitive elements within known deafness genes. Segdups, defined as regions with > 95% homology, are features that are causally implicated in recurrent CNVs mediated by NAHR and were identified in seven deafness genes: *TSPEAR* (4 segdups), *OTOA* (1), *STRC* (1), *MYO3A* (1), *ESPN* (1), *OTOGL* (1), *CACNA1D* (1). Both *OTOGL* and *CACNA1D* have homology to regions on different chromosomes, which predisposes to inter-chromosomal translocation via NAHR.

Alu and L1 elements are smaller repetitive elements and predispose to recurrent NAHR and non-recurrent NHEJ. Only six genes on our panel lacked Alu or L1 elements, and when we normalized the number of these elements by gene size, we found significantly more Alu elements, L1 elements, and total Alu + L1 elements in CNV-associated genes (paired *t*-test, *P* = 0.0003, *P* = 0.0104, *P* = 0.0139, respectively; Additional file 3). In contrast, a comparison of all simple repeat elements normalized per gene found no significant difference between the 16 CNV-associated genes and the remaining genes on our panel (*P* = 0.6442), suggesting that the increased repetitive element burden could explain, at least in part, the gene distribution of CNVs we observed.

To illustrate the impact of CNV identification in the genetic evaluation of NSHL, we present three representative cases below.

### Biallelic conversion of the *STRC* gene

Patient 45 was evaluated for mild sensorineural hearing loss (SNHL) after failing the newborn hearing screen (NBHS). A diagnostic auditory brainstem response (ABR) at 4 weeks showed bilateral mild hearing loss and the patient was fitted with binaural amplification. There was no family history of hearing loss.

**Figure 1:**
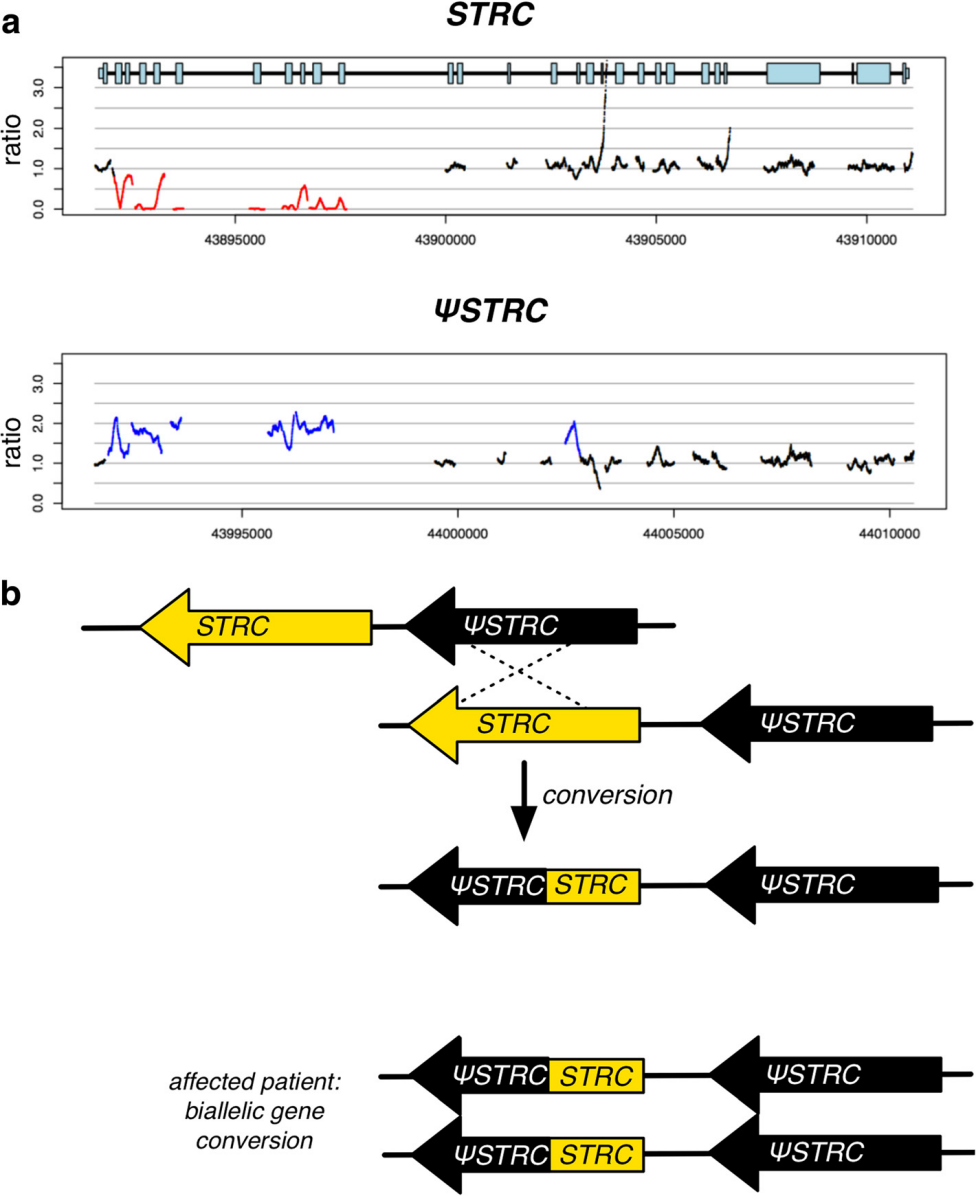
**A biallelic gene-pseudogene conversion of *STRC *is the causative mutation in patient 45. (a)** Ratio plots showing apparent homozygous deletion of first 11 exons of *STRC* and duplication of first 11 exons of ψ*STRC*. **(b)** Hypothesized mechanism of gene-pseudo-gene conversion (non-allelic gene conversion *in trans*) and depiction of the biallelic change in the patient.

TGE-MPS identified a biallelic partial gene conversion involving *STRC* and the pseudogene, ψ*STRC*. As shown in Figure 1, the last 11 exons of *STRC* have been converted to ψ*STRC* leading to a functionally null *STRC*. This change represents the first report of a biallelic gene-pseudogene conversion at this locus. *STRC* encodes stereocilin, which localizes to the tips of outer-hair-cell stereocilia where it is hypothesized to form tip-link connectors between stereocilia as well as part of the connection between outer hair cells and the tectorial membrane [25]. Its mutation causes mild hearing loss. Additionally, because the *STRC* gene region is associated with a segdup that results in pseudogenes of both *STRC* and *CATSPER2*, homozygous deletions of the entire region (*STRC* and *CATSPER2*) cause DIS, characterized by mild SNHL in males and females and sperm motility defects and infertility in males [7].

In our cohort of 686 patients, we identified causative mutations in *STRC*, including homozygous deletions, gene conversions, and heterozygous deletions *in trans* to a missense change, in 5.4% of patients (37/686; Table 2), accounting for 13.8% of all genetic diagnoses we provided (37/267) and making mutations in *STRC* one of the most common causes of ARNSHL, following closely behind mutations in *GJB2* in primarily Caucasian populations. The carrier frequency for deletions, conversions, and duplications at the *STRC* locus was 4.7% (65/1,372 alleles), 2.6% (35/1,372 alleles), and 0.4% (5/1,372 alleles), respectively.

Forty-six patients carried at least one deletion in the *STRC* gene region (Additional file 2). We were able to determine the status of *CATSPER2* and ψ*STRC* in 13 of these patients because they were tested with OtoSCOPE v5 (Materials and methods; Additional file 1). We found that the *CATSPER2* gene was involved in 77.0% (10/13) of these patients, including one individual in which the deletion encompassed only *CATSPER2* and ψ*STRC* and not *STRC*. Four individuals were homozygotes for a large contiguous *STRC-*ψ*STRC-CATSPER2* deletion, indicating that they in fact are affected by DIS and not NSHL. All four of these patients are female and therefore will not have decreased fertility.

### Deletion of *OTOA* in a Caucasian individual

Patient 14 was diagnosed with congenital moderate-to-severe SNHL after failing the NBHS, tested with otoacoustic emissions. A diagnostic ABR at 6 weeks confirmed the hearing loss and the patient was fitted with binaural amplification. There was no family history of hearing loss. The patient’s ethnicity was European-American.

**Figure 2:**
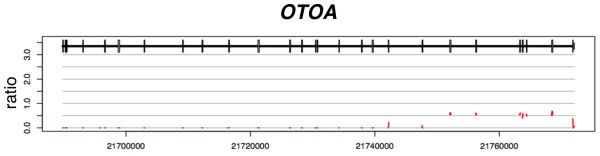
**Ratio depth-of-coverage plot showing homozygous deletion of *OTOA *as the causative mutation in patient 14.** The last 10 exons appear to be only heterozygous deletion, but this is an artifact due to segmental duplication of these exons (see text for details).

TGE-MPS identified a homozygous deletion of the entire *OTOA* gene and the patient was diagnosed with DFNB22-related hearing loss (Figure 2). *OTOA* encodes otoancorin, an extracellular membrane protein that localizes to the interface between the tectorial membrane and the sensory epithelium [26]. It contains a glycophosphatidylinositol anchor that is thought to mediate attachment between the tectorial membrane and the greater epithelial ridge and spiral limbus during development, and with the spiral limbus in the mature cochlea. Mice lacking *Otoa* have hearing loss secondary to an abnormality of the tectorial membrane, which is attached at to the outer hair cells but detached from the spiral limbus [27].

To date, three mutations in *OTOA* have been reported within the Palestinian population: IVS12(+2)T > C [26], c.1067A > T, p. D356V [28], and a 500 kb deletion [29]. Two pathogenic *OTOA* mutations were recently discovered in the Pakistani population: p.G451D and p.P627S [30]. The deletion we report here is therefore the first report of a mutation in *OTOA* in a person of non-Middle-Eastern ethnicity. We identified 18 CNVs involving *OTOA*, including 15 deletions, 3 conversions and 3 duplications, making CNVs of *OTOA* the second most commonly identified CNVs after *STRC*. This frequency likely reflects the fact that exons 20 to 28 are part of a segdup of > 99% identity located 820 kb away [29]. In five cases, including this example, the CNV was causative.

### A two exon deletion in *TMC1*

**Figure 3:**
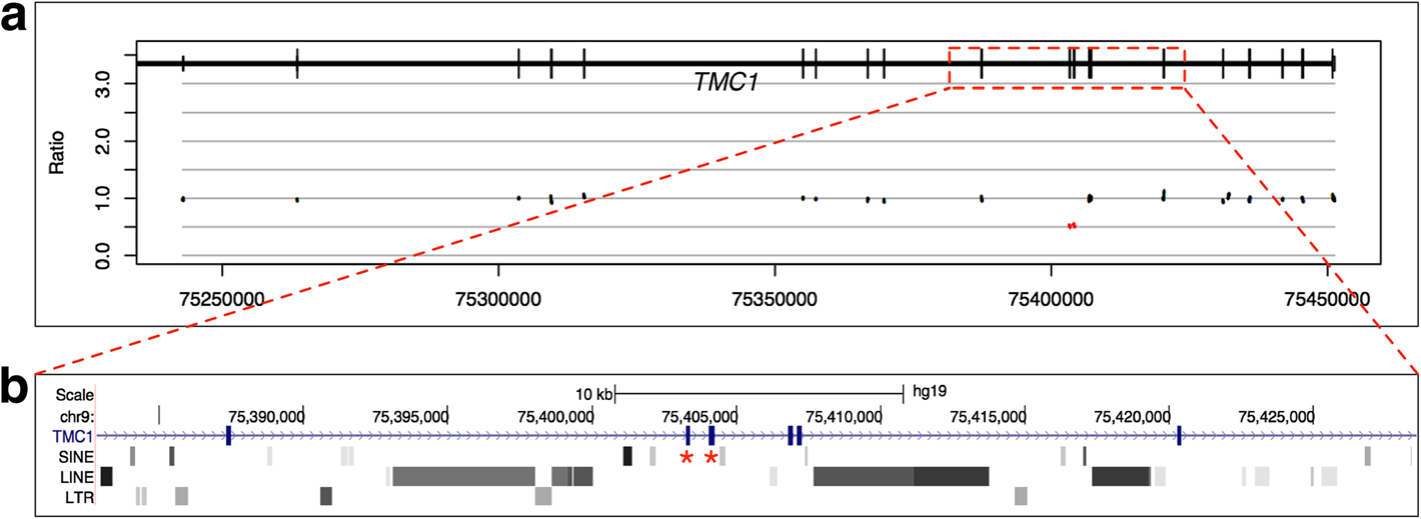
**A two-exon deletion of *TMC1 *is responsible for deafness in patient 100. (a)** Ratio depth-of-coverage plot showing a two-exon heterozygous deletion. This deletion was found *in trans* with a missense change predicted to be pathogenic (c.1276G > A, p.Ala426Thr). **(b)** Highlighted region from UCSC genome browser RepeatMasker track demonstrates multiple short interspersed elements (SINEs; primarily Alu repeats), long interspersed elements (LINEs; including L1 repeats), and long terminal repeats (LTR) in this region. The two deleted exons are marked with asterisks. The hypothesized mechanism for this deletion involves these repeat elements and NAHR.

Patient 100 failed the NBHS and was subsequently diagnosed with profound, symmetric NSHL by follow-up ABR. There was no reported family history of hearing loss. TGE-MPS identified an incidental heterozygous change in *GJB2* (c.101 T > C p.M34T) and a heterozygous two-exon deletion (exons 14 and 15) of *TMC1* (Figure 3). Although the precise breakpoints could not be determined, the CNV encompasses the entirety of exons 14 (145 bp) and 15 (195 bp) and intron 14, making it between 1 and 20 kb in size (Figure 3). The deletion is out of frame and segregates opposite a c.545G > A p.Gly182Asp missense change in exon 11. Pathogenicity software shows this missense change to be damaging or deleterious by six *in silico* scoring methods (SIFT, PhyloP, Polyphen, LRT, MutationTaster, and GERP; see [9] for details) and thus is predicted to be pathogenic. The variant has a reported frequency in the Exome Variant Server of 0.000154.

Mutations in *TMC1* cause ARNSHL at the DFNB7/11 loci and ADNHSL at the DFNA36 locus [31]. More than 35 mutations have been reported as deafness causing [32,33], including two large deletions leading to DFNB7/11-related hearing loss [31,34]. This patient therefore carries the third deletion of this gene to be associated with NSHL and is the first report of a hemizygous deletion opposite a missense mutation.

## Discussion

In this study, we performed comprehensive genetic testing of 686 patients with NSHL and showed that CNVs are a common contributor to hearing loss. We identified 143 CNVs in 16 deafness genes, including in 7 genes in which CNVs have not been reported (Table 1). Nearly one in three deafness genes (25 of 89 genes, 28%) carried a CNV and in aggregate CNVs contributed to nearly one in five (18.7%) genetic diagnoses for NSHL. These results mandate CNV screening in all comprehensive genetic testing platforms for deafness. Ideally, CNV detection should be incorporated in the diagnostic pipeline as a single test, which we accomplished by integrating a customized read-depth approach for variant detection to our TGE-MPS platform.

NAHR, the primary mechanism of formation of recurrent CNVs, is generally mediated by segdups or low-copy repeats [2]. Segdups range in size from 10 to 300 kb and typically must have > 95% homology for recombination to occur. Depending on the orientation of the segdup, an NAHR event can lead to duplications and deletions (segdups in parallel), inversions (segdups in opposition), or translocations (segdups on different chromosomes). NHEJ and FoSTeS, in comparison, are mediated by smaller repetitive elements, including short interspersed elements (SINES) such as Alu elements, and long interspersed elements (LINES) such as L1 repeats. Because NHEJ is dependent on repair of double-strand breaks, it leaves a signature ‘scar’ at the point of the repair. FoSTeS occurs during DNA replication and causes deletions, duplications and, when multiple FoSTeS events occur in sequence, complex rearrangements. ‘Joint points’ , repetitive elements and regions of microhomology of 2 to 3 bp are often the sites of FoSTeS.

The greatest number of CNVs was identified in *STRC* and *OTOA*, comprising 73% and 13% of all CNVs identified, respectively (Table 2). These two genes contain NAHR-predisposing segdups. We also identified segdups in *TSPEAR*, *MYO3A*, *ESPN*, *OTOGL* and *CACNA1D*, five genes in which CNVs have not been reported. In genes carrying CNVs in this study, we found a significantly higher number of small repetitive elements. We also identified several genes highly enriched for repetitive elements and thus likely candidates for CNVs (*MARVELD2*, *CCDC50*, *LHFPL5*, *DIABLO*, *SLC26A5*; see Additional file 3 for a full list).

Although our TGE-MPS platform precludes the determination of breakpoints, we obtain outstanding exon-level resolution for CNV detection and can identify CNVs, single nucleotide changes and indels simultaneously. By tiling probes across pseudogenes in segdups we are able to reliably determine gene conversions and thereby improve our diagnostic ability.

The carrier frequency of the most common CNV, a deletion of the *STRC* region, was 4.7% in this study. Previous studies of patients with hearing loss using less sensitive SNP arrays for CNV detection have found carrier frequencies of 1.8% (659 patients) [6] and 6.4% (94 patients) [35]. Two other studies using array CGH reported a carrier frequency in individuals without hearing loss undergoing genetic testing for other reasons to be 1.1% and 1.6% [23,36]. These data suggest that, in some populations, the carrier frequency of deletions of the *STRC* region may be more common than the carrier frequency of the most common mutations in *GJB2*. However, it is also important to consider the potentially pathogenic gene-pseudogene conversions at this locus. In this study we found the carrier frequency of these conversions to be 2.6%. Other platforms may not be able to accurately detect these potentially pathogenic conversions and so the carrier frequency for deleterious CNVs of this region is most likely under-reported.

Deletions of the *STRC* region are especially important to detect because concurrent deletion of the adjacent *CATSPER2* gene leads to DIS in males [7]. In those patients for whom we used the modified version of our panel that includes the *CATSPER2* gene, we identified its involvement in 10/13 cases with *STRC* deletion, suggesting that DIS is likely an under-diagnosed cause of hearing loss and male infertility.

This study has three minor limitations. First, the samples we studied were submitted for clinical testing and therefore detailed family history, clinical and/or phenotypic data were not available, precluding robust genotype-phenotype correlations and decreasing certainty of pathogenicity for CNVs identified. Second, we did not evaluate the carrier frequency of these CNVs in a control population, which remains an area of future study. And last, we used TGE-MPS for CNV detection, making it impossible to map breakpoints that occur outside targeted exonic regions. This constraint means that if a CNV spans multiple genes, it is impossible to determine its approximate size. However, when commonly occurring CNVs are recognized, additional probes can be added to any TGE-MPS platform to increase information content. For example, we have included more probes for the *STRC/CATSPER2* region in recent versions of the platform used here.

## Conclusion

CNVs are a common cause of NSHL. Their involvement in one in five genetic diagnoses by our laboratory using a comprehensive genetic testing platform mandates their identification in any clinical genetic diagnostic test for deafness. These data suggest that CNVs are likely to play an important role in other genetic disorders as well.

## Abbreviations

ABR: auditory brainstem response; ADNSHL: autosomal dominant non-syndromic hearing loss; ARNSHL: autosomal recessive non-syndromic hearing loss; bp: base pair; CGH: comparative genomic hybridization; CNV: copy number variant; DIS: deafness-infertility syndrome; FoSTeS: fork stalling and template switching; MLPA: multiplex ligation-dependent amplification; MPS: massively parallel sequencing; NAHR: non-allelic homologous recombination; NBHS: newborn hearing screen; NHEJ: non-homologous end joining; NSHL: non-syndromic hearing loss; SNHL: sensorineural hearing loss; TGE: targeted genomic enrichment.

## Competing interests

The authors declare that they have no competing interests.

## Authors’ contributions

Designed study: AES, DLK, HA, RJHS. Performed experiments: KLF, ETC, CJN. Performed analysis: AES, DLK, HA, CMS, CJN, EAB-Z. Collected and assembled data: AES, DLK, KLF, AEW, CJN. Drafted manuscript: AES, RJHS. All authors contributed to, edited and reviewed the final manuscript.

## Supplementary Material

Additional file 1: Table S1 Genes sequenced in OtoSCOPE v4 and v5. Click here for file

Additional file 2: Table S2 Detailed information on CNVs identified in this study. Click here for file

Additional file 3: Table S3 Repetitive element burden in known hearing loss genes. Click here for file
